# Clinical validation of controlled grass pollen challenge in the Environmental Exposure Unit (EEU)

**DOI:** 10.1186/s13223-015-0071-3

**Published:** 2015-01-27

**Authors:** Anne K Ellis, Lisa M Steacy, Barnaby Hobsbawn, Caroline E Conway, Terry JB Walker

**Affiliations:** Division of Allergy & Immunology, Department of Medicine, Queen’s University, Kingston, ON Canada; Allergy Research Unit, Kingston General Hospital, 76 Stuart Street, Kingston, ON K7L 2 V7 Canada

**Keywords:** Allergic rhinitis, Environmental exposure unit, Grass pollen, Controlled allergen challenge

## Abstract

**Rationale:**

The Environmental Exposure Unit (EEU), a controlled allergen exposure model of allergic rhinitis (AR), has traditionally utilized ragweed pollen. We sought to clinically validate the use of grass pollen in the EEU.

**Methods:**

Healthy volunteers with a history of AR symptoms during grass pollen season and supportive skin test responses attended the EEU for 3 hrs of rye grass pollen exposure (*Lolium Perenne*). Non-atopic controls were also recruited. Participants assessed individual rhinoconjunctivitis symptoms to generate Total Nasal Symptom Score (TNSS; max 12) and Total Symptom Score (TSS; max 24) and recorded Peak Nasal Inspiratory Flow (PNIF) q30min while in the EEU. Participants returned the following day for an additional 3 hrs of pollen exposure. Two separate groups allowed for the exploration of lower vs. higher pollen concentrations and subsequent effects on symptoms.

**Results:**

78 participants were screened, of whom 39 were eligible and attended the 2x3h EEU visits, plus 8 non-atopic controls. Mean TSS, TNSS and PNIF values amongst participants in the higher pollen concentration group (target 3500 grains/m3) after the first 3 hr exposure were 18.9, 9.7 and 68 L/min, respectively. In comparison, mean TSS, TNSS and PNIF values in the lower pollen concentration (2500 grains/m3) group were only 13.3, 7.6, and 82 L/min, respectively. The subsequent day of pollen exposure did not appreciably alter the maximal TSS/TNSSs, but rather resulted in a more rapid onset of symptomatology, with higher mean scores at the 30 min, 60 min and 90 min timepoints. The non-atopic controls remained asymptomatic.

**Conclusions:**

This study provides clinical validation of the ability to generate allergic rhinoconjunctivitis symptoms amongst grass-allergic individuals in the EEU.

## Introduction

A significant proportion of the North American population suffers from the symptoms of seasonal allergic rhinitis (SAR) [1]. The Environmental Exposure Unit (EEU), developed at Kingston General Hospital and Queen's University in Kingston, Ontario, Canada, was the first such facility to be developed in North America, and is an internationally recognized and validated controlled allergen challenge model of allergic rhinitis (AR). This specially designed room provides a unique study environment ideally suited to evaluate various pathophysiologic processes at work in AR, as well as to assess the efficacy and onset of action of various anti-allergic medications [2].

The EEU is a room measuring 21 × 15 m with a feeder system that delivers controlled levels of commercially obtained pollen into the seating area. Fans are organized to continuously circulate the air in the room, and seven impact type particle samplers (Rotorod® counters) are arranged to determine pollen levels throughout the room every 30 minutes. This particular sampling technology has been studied and shown to produce optimal pollen measurements within a controlled allergen challenge facility [3]. The pollen emission rate is modified based on these counts to maintain pollen concentrations within a narrow range. Thus, the EEU provides a closed environment in which participants are exposed to a predetermined, controlled, and constant level of airborne pollen.

The EEU has proven effective in multiple studies evaluating various aspects of seasonal allergic rhinitis and its treatments [4-17]. Previous studies conducted in the EEU using ragweed pollen have shown 3500 grains/m^3^ ± 500 to be an optimal concentration to produce symptoms similar to those experienced in the outdoor environment. All previous EEU clinical trials have utilized ragweed pollen as the allergen of choice for dispersal, due to the frequency of ragweed allergy in the local population. Many emerging treatments for allergic rhinitis, however, are allergen specific (e.g. novel immunotherapeutics), therefore it was desirable to expand the repertoire of pollen selection for use in the EEU.

**Figure 1:**
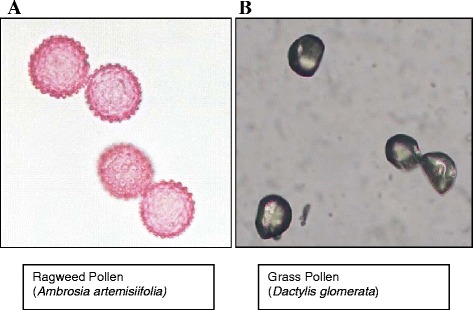
**Microscopic representation of a typical ragweed pollen (A) and grass pollen (B) grain.** Notably, ragweed pollen has a burr-like surface with tiny spikes on its surface, compared with the smooth surface of grass pollen.

Each type of pollen has unique features; ragweed pollen has a barbed, “spiky” appearance on surface analysis, while grass pollen has a smooth, almost pearl-like surface (Figure 1). The barbed surface area of the ragweed pollen gives it a “sticky” characteristic, increasing the rate of coincidence of pollen grains as experienced while viewing the Rotorod ® air samples under the microscope. This coincidence, or sticking-together, of pollen grains may also improve the pollen’s ability to stay aloft during increased air current velocities.

Grass pollen has a particle size of 30 to 39 microns (as compared to 18 to 20 for ragweed), which could potentially either hinder or increase its ability to be suspended. With enough air current to overcome its size and increased mass, the large surface area of the grass pollen would allow it to be positively affected by the air current’s ability to keep it aloft. This loftiness may also promote the grass pollen’s ability to travel longer distances without “falling out” of the air stream. Essentially, both pollens have characteristics which promote the ability to keep the pollen grains suspended and hence, the ability to maintain proper concentrations within the EEU. Preliminary validation studies conducted in the EEU while it was fully set up, but absent of human volunteers, have confirmed our ability to release, disperse, and maintain grass pollen concentrations in the EEU utilizing the existing pollen dispersal technology [18].

In order to explore and determine the clinical validity of grass pollen delivery in the EEU, two concentrations of grass pollen were compared to evaluate the potential impact these differences may have on symptom scoring: 3500 grains/m^3^ and 2500 grains/m^3^. This study aimed to determine the optimal amount of grass pollen/m^3^ required to ensure participants experience allergic symptoms in the EEU similar to those they would experience outdoors, and additionally to clinically validate the use of grass pollen within the EEU. Of note, despite the rapid increase in the development of several controlled allergen challenge facilities (CACF) as a model of allergic rhinitis, to date, no international standards have been established for validation of such facilities.

## Methods

This study protocol was reviewed and approved by Queen’s University and Affiliated Teaching Hospitals Research Ethics Board, and all participants gave written informed consent prior to the initiation of any study related procedures.

### Participants

Participants on file were approached to participate in the current study. Eligible participants were males or females between the ages of 18 and 65 with a positive skin prick test to rye grass allergen at screening—with a wheal diameter at least 3 mm larger than that produced by the negative control—who were able and willing to provide written informed consent. Females of childbearing potential were required to use a medically acceptable method of birth control or to be abstinent throughout the study. Participants unable to adhere to the washout periods for the medications listed in Table 1 prior to screening or pollen exposure visits, as well as those who were experiencing an upper respiratory tract infection within one week of any of the visits, were excluded. Also ineligible were participants with asthma requiring the use of a short-acting beta agonist greater than twice a week, and those with a history of any disease or disorder that, in the judgement of the investigator, would impact a participant’s safety or the results of the study. Participants with a known history of positive test results for Hepatitis B, Hepatitis C, HIV or tuberculosis other than what would be anticipated following vaccination, or currently receiving allergen specific immunotherapy injections were excluded.

**Table 1:** **Medication washout periods**

**Medication class**	**Washout period**
Antihistamines	7 days
Intranasal or inhaled corticosteroids	14 days
Intranasal or inhaled cromolyn	14 days
Systemic corticosteroids or astemizole	30 days

Participants were required to adhere to these washout periods prior to screening or pollen exposure visits.

### Study design

Participants initially attended a screening visit where the following procedures were conducted: vital signs, skin testing to a panel of allergens (rye grass, timothy grass, standardized grass mix, ragweed, birch, tree mix, dog, cat, *D. pteronyssius* (dust mite), *D. farinae* (dust mite), and *Alternaria*), height and weight, physical examination including detailed nasal examination, and urine pregnancy testing (for women of childbearing potential only).

Eligible participants returned to the EEU for two 3 hour sessions of rye grass pollen (*Lolium Perenne*) exposure occurring on subsequent days. Participants were randomly divided into two groups, attending separate two-day sessions in the EEU. The two groups were exposed to different levels of target grass pollen concentration in the EEU, either a lower pollen concentration of 2500 ± 500 grains/m3 or a standard pollen concentration of 3500 ± 500 grains/m3. Peripheral blood samples were collected before and after the visit (results not reported here), and participants were asked to record their symptoms on diary cards at the beginning of the session, and every half-hour throughout the session. Symptoms included rhinnorhea, nasal congestion, sneezing, nasal itching, itchy/watery eyes, red/burning eyes and itching of the ears/palate/throat (refer to Table 2 for definitions). These cards were scanned electronically. The individual rhinoconjunctivitis symptom scores submitted on the diary cards were tallied to create a Total Nasal Symptom Score and a Total Symptom Score (max of 12 and 24 respectively, see Table 2) for each participant. Additionally, participants measured their Peak Nasal Inspiratory Flow (PNIF) using the In-Check PNIF meter (Clement Clark International) at baseline and every 30 minutes during pollen exposure.

**Table 2:** **Symptom score definitions**

**Rating**	**Definition**
0 = None	Symptom is completely absent
1 = Mild	Symptom is present, but not bothersome
2 = Moderate	Symptom is bothersome, but tolerable
3 = Severe	Symptom is hard to tolerate, desiring treatment

Participants used this rating system to score their symptoms of rhinnorhea, nasal congestion, sneezing, nasal itching, itchy/watery eyes, red/burning eyes, and itching of the ears/palate/throat. The individual symptom scores were tallied to create a Total Nasal Symptom Score (max 12) and a Total Symptom Score (max 24) for each participant.

Participants remained in the EEU for the duration of the 3 hour pollen exposure period unless, in the judgement of the investigator, symptoms became so intolerable that removal from the EEU was warranted. Upon leaving the research centre, participants were provided with a package of diary cards in order to continue recording their symptoms and PNIF on an hourly basis, until 12 hours had passed since the onset of the pollen exposure.

The next day, participants returned to the EEU for the second of the two 3 hour sessions of grass pollen exposure, and again recorded their symptom scores and PNIF measurements every 30 minutes. The purpose of this second day visit was to investigate the potential for any priming of responses on Day 2 compared to Day 1, and to evaluate tolerability of two “back to back” exposures.

### EEU methodology

**Figure 2:**
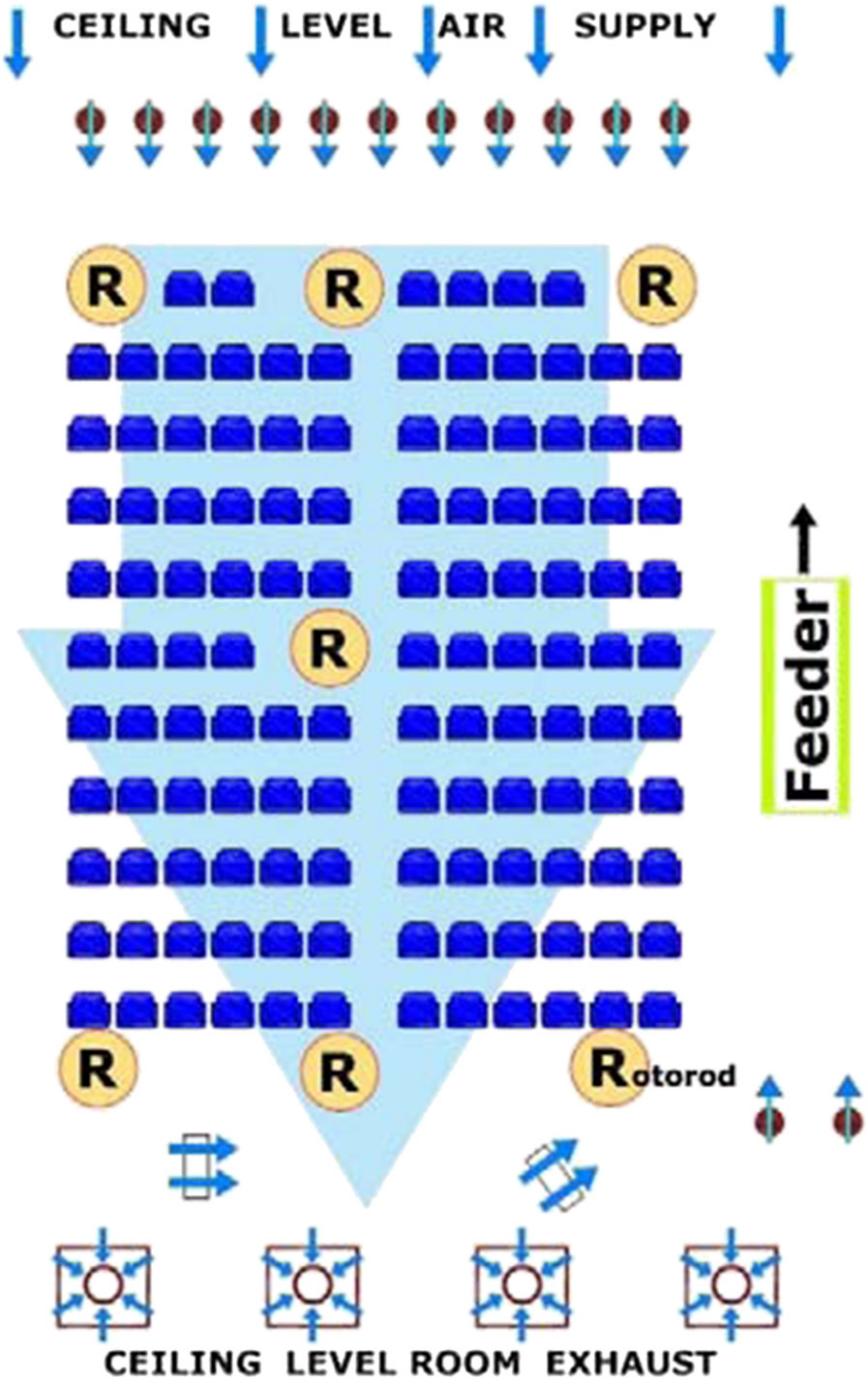
**Layout of the Environmental Exposure Unit (EEU).**

As described above, the Kingston EEU is a specifically engineered room located within Kingston General Hospital. The EEU setup, including location of chairs, feeder, fans, and Rotorod® sampling equipment, is illustrated in Figure 2. A custom engineered computer and laser-aided system disperses pollen, at a single point of delivery (Figure 2). The pollen is propelled, by selectively placed groups of directional fans, over the participant seating area. For the current study, rye grass pollen was sourced from Greer Laboratories (Lenoir, NC), and was independently tested for fungal and bacterial contamination (Paracel Laboratories, Ottawa, ON, Canada) prior to its use.

### Statistical analysis

In order to discern correlations between reported measures of nasal congestion, TNSS, and PNIF for each participant, Pearson’s correlation tests were conducted. The statistical software GraphPad Prism 6 (GraphPad Software, Inc) was used.

## Results

78 potentially grass-allergic participants were screened, of whom 39 were eligible and attended the 2 × 3 hour EEU visits. 20 grass-allergic people attended the visits with the higher pollen concentration and 19 were in the lower target pollen concentration group. Following screening, 8 eligible non-atopic controls were similarly enrolled. The non-atopic controls remained asymptomatic throughout both days of pollen exposure in both groups.

### Total Symptom Score (TSS) and Total Nasal Symptom Scores (TNSS)

**Figure 3:**
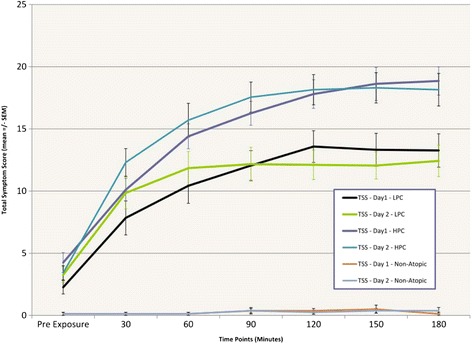
**Total Symptom Scores (TSS) were higher in the higher pollen concentration group (HPC) than the lower pollen concentration group (LPC) after three hours of pollen exposure.** The second day of pollen exposure in both groups resulted in similar peak TSSs after 3 hours of exposure as the day previous, but generated a more rapid increase in TSS.

**Figure 4:**
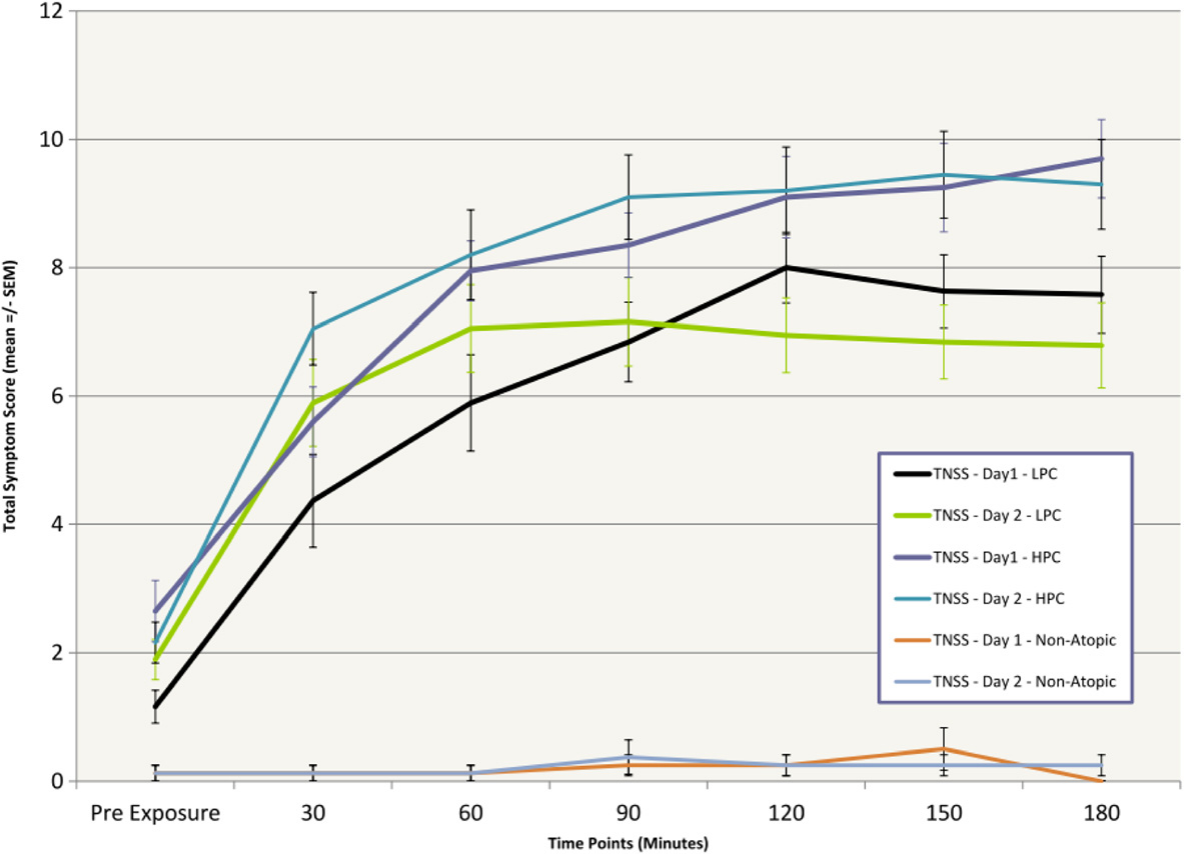
**Total Nasal Symptom Scores (TNSS) were higher in the higher pollen concentration group (HPC) than the lower pollen concentration group (LPC) after three hours of pollen exposure.** Similar to TSS, final TNSSs on Day 2 were similar to those reported on Day 1 after 3 hours of pollen exposure, however, scores increased more quickly on Day 2.

Mean TSS and TNSS scores obtained in both the higher (target 3500 grains/m3) and lower (2500 grains/m3) pollen concentration groups over both days of exposure are summarized in Figures 3 and 4. Mean TSS and TNSS amongst allergic participants in the higher pollen concentration group after the first 3 hr exposure (i.e. at hour 3) were 18.9 (SD = 2.7) and 9.7 (SD = 5.1), respectively. In comparison, mean TSS and TNSS values in the lower pollen concentration group were only 13.3 (SD = 5.9) and 7.6 (SD = 2.6), respectively.

The subsequent day of pollen exposure did not appreciably alter the maximal TSS/TNSSs in either group, but rather resulted in a more rapid onset of symptomatology, with higher mean scores at the 30 min, 60 min, and 90 min time points. Specifically, after 30 min of pollen exposure on Day 2, the TSS in the higher concentration group was 12.3 (SD = 5.0)—compared to 10.1 (SD = 4.1) on Day 1, and the TNSS reached 7.1 (SD = 2.5)—compared to 5.6 (SD = 2.4) on the previous day. Earlier rises in symptom scores were also observed in the lower pollen concentration group on Day 2. After 30 min of grass pollen exposure, the TSS and TNSS scores rose to 9.8 (SD = 5.6) and 5.9 (SD = 3.0) respectively on Day 2, compared to the mean scores of 7.8 (SD = 6.0) and 4.4 (SD = 3.2) obtained the day before.

### Peak nasal inspiratory flow

PNIF measurements obtained both at baseline and at each pollen exposure time point were lower in the high pollen concentration group than in the low concentration group. For example, after the first 3 hours of pollen exposure, the higher concentration group reported a mean PNIF value of 68.1 L/min (SD = 28.4), compared to 82.4 L/min (SD = 43.6) in the lower concentration group.

PNIF measurements dropped notably from baseline in both pollen concentration groups after 3 hours of grass pollen exposure. At hour 3 of their first EEU visit, the mean PNIFs of the higher and lower concentration groups were 29.8 L/min (relative change of 30.4%) and 42.9 L/min (34.2%) less than those reported at baseline, respectively. Similar to TSS and TNSS, the second day of pollen exposure did not result in very different changes in PNIF from baseline, but resulted in a more rapid drop in recorded PNIF measurements. After 30 min of pollen exposure, the mean PNIF fell by 22.8 L/min (26.1%) in the higher concentration group on Day 2, compared to a 12.9 L/min (13.1%) drop on Day 1. 30 min of pollen exposure also resulted in a quicker drop in mean PNIF in the lower concentration group, which reported a change of 19.5 L/min (16.9%) from baseline on Day 2 compared to a 10.3 L/min (8.2%) drop the previous day. As well, participants in both pollen exposure groups reported a lower mean baseline PNIF measurement at the beginning of their second day of pollen exposure than the day before.

On both days of pollen exposure in each concentration group, a significant low to moderate inverse correlation was observed between a participant’s subjective scoring of nasal congestion and their mean PNIF measurements. After performing a correlative analysis as described previously, r-values for Days 1 and 2 in the high pollen concentration group and Days 1 and 2 in the low group were −0.3224, −0.3457, −0.3753, and −0.3390, respectively (p < 0.0001 for all). A significant positive correlation was also observed between reported measures of nasal congestion TNSS scores. R-values for Days 1 and 2 in the high pollen group and Days 1 and 2 in the low pollen group were 0.8667, 0.8663, 0.8952, and 0.8156, respectively (p < 0.0001). In addition, comparing the standard deviations of the data obtained in both the high and low pollen concentration groups, there was no appreciable difference in the variability of data between the two days.

## Discussion

Exposure of timothy grass skin test positive subjects with a clinical history of SAR symptoms during grass pollen season (over at least 2 consecutive past seasons) to controlled levels of rye grass pollen in the EEU resulted in the development of typical symptoms of SAR in a reproducible manner. This serves as further confirmation of the extensive clinical cross-reactivity amongst grass pollens. Using a lower target pollen concentration of 2500 grains/m3 corresponded with a lower mean symptom burden compared to 3500 grains/m3. Upon return to the EEU the following day for a subsequent 3 hour exposure to the same target pollen concentration, maximal TSS and TNSS responses were not appreciably higher by the 3 hour time point, but rather these maximal responses were achieved sooner, allowing for more predictable responses. Similarly, changes in PNIF measurements from baseline in response to pollen exposure were more rapid in participants from both groups on the second day of exposure. This second day of exposure was included to evaluate this ‘priming’ effect which has been noted previously in EEU studies [2], whereby symptoms develop more rapidly upon subsequent exposures. Additionally, it served to determine whether or not back to back exposures led to more severe symptom scores, which did not in fact appear to be the case.

Mean PNIF measurements obtained throughout the duration of pollen exposure were lower in the higher pollen concentration group, likely reflecting more severe nasal symptoms being produced in response to the 3500 grains/m3 concentration compared to the 2500 grains/m3 concentration. This conclusion is supported as well by the higher TNSS and TSS responses reported by the higher concentration group. As expected, the participant’s subjective scoring of nasal congestion correlated well with PNIF measurements, as well as TNSS, with increased congestion presumably obstructing nasal inspiration and resulting in the lower PNIF values.

Participants in both pollen groups reported lower baseline PNIF measurements on their second visit to the EEU. This suggests that grass pollen exposure, and thus SAR symptoms generated from the previous day, continues to affect PNIF prior to a second exposure to grass pollen. This result suggests a generation of a late phase response in a proportion of the participants, and is consistent with historically established symptom patterns of SAR.

The symptom scores generated from the group exposed to 3500 grains/m^3^ of grass pollen generally mirrored the responses seen previously with our ragweed pollen exposures, with some individuals reacting quickly with high symptom scores and others to a lesser degree [8]. The overall degree of symptomatic responses was also similar to those seen in ragweed sensitive subjects in a study conducted to evaluate the presence or absence of late phase allergic rhinitis responses to ragweed challenge in the EEU [19]. In both of these studies, the mean TNSS at hour 3 was 8.6 to 9.2 with standard deviations of 2.0-2.1, respectively (8, 19, data on file). The predictability of more rapid allergic symptoms achieved on the second day of pollen exposure may help to control for this variability, though no meaningful change in standard deviations between Day 1 and Day 2 data in the high or low concentration groups was observed.

This study confirms that, similar to ragweed pollen, grass pollen dispersed in the EEU can be effectively used to elicit AR symptoms in grass-allergic individuals. Thus, the EEU can be reliably used to study AR in grass-allergic participants, increasing both the scope of utilization of the unit and the pool of available participants. In addition, such that both allergens have now been successfully validated, the difference in symptomology of ragweed and grass-triggered AR may be discerned in patients allergic to both allergens, as can the efficacy of interventions targeting each specific allergy. Other CACF have developed the capabilities to disperse several different types of pollen, although not all have published their validation work in the searchable literature. Of note, the Biogenics Research Chamber in San Antonio, Texas, however, recently published a very interesting study showing the symptomatic responses of grass allergic subjects exposed to Timothy grass in the CACF, despite the fact that the participants were recruited from a geographic area where Timothy grass does not grow [20].

By examining the different responses elicited by two different concentrations of grass pollen, the current study has also produced a standardized framework for subsequent grass-specific EEU trials. The higher symptom burden triggered in individuals exposed to 3500 grains/m3 of pollen compared to 2500 grains/m3 would be better suited to study the effects of the disease, as well as provide a wider range to study the degree of symptomatic improvement achieved by AR treatments. Thus, we have defined 3500 grains/m3 as the optimal grass-pollen concentration to be used as a standard for future EEU trials by confirming its ability to produce appropriate AR symptoms, the clinical validation of this method supporting the results of future studies.

## Conclusions

Exposure to grass pollen in the EEU produces reliable symptoms of allergic rhinitis in grass-sensitive patients. The EEU can therefore be used to conduct clinical trials evaluating grass-specific allergic interventions.

## Abbreviations

AR: Allergic rhinitis
EEU: Environmental Exposure Unit
PNIF: Peak Nasal Inspiratory Flow
SAR: Seasonal allergic rhinitis
TNSS: Total Nasal Symptom Score
TSS: Total Symptom Score
